# MDSCs are involved in the protumorigenic potentials of GM-CSF in colitis-associated cancer

**DOI:** 10.1177/0394632017711055

**Published:** 2017-05-23

**Authors:** Ning Ma, Qilin Liu, Lin Hou, Yalin Wang, Ziling Liu

**Affiliations:** 1 Department of Rheumatology, First Affiliated Hospital, Jilin University, Changchun, P.R. China; 2 Department of Oral and Maxillofacial Surgery, Hospital of Stomatology, Jilin University, Changchun, P.R. China; 3 Cancer Center, First Affiliated Hospital, Jilin University, Changchun, P.R. China

**Keywords:** GM-CSF, CAC, MDSC

## Abstract

Chronic inflammation is thought to be a major driving force for the development of colitis-associated colorectal cancer (CAC). As one member of proinflammatory cytokine family, granulocyte macrophage colony-stimulating factor (GM-CSF) has been identified to play a key role in CAC pathogenesis recently. The underlying mechanisms, however, remain largely unknown. In this study, we found that myeloid-derived suppressor cells (MDSCs) accumulated increasingly in the lesions during the progression from colitis to cancer, which was critical for CAC formation. Importantly, this MDSC accumulation was controlled by GM-CSF. MDSC number decreased significantly in GM-CSF-deficient mice suffering from CAC induction, and transfusion of MDSCs from wild-type CAC-bearing mice into GM-CSF-deficient counterparts led to recurrence of CAC. Furthermore, the supernatants of CAC lesions or GM-CSF alone was sufficient to differentiate hematopoietic precursors into MDSCs. Addition of neutralizing anti-GM-CSF antibody impaired the MDSC-differentiating effects of the supernatants of CAC lesions. Overall, these findings shed new insights into the mechanisms of GM-CSF underlying CAC development, by inducing/recruiting CAC-promoting MDSCs. Blocking GM-CSF activity or MDSC function may represent new therapeutic strategies for CAC in clinic.

## Introduction

It is well established that chronic unresolved inflammation represents a major driving force for the initiation and progression of malignancies in many organs.^1^ For instance, in the patients suffering from ulcerative colitis, the risk of colorectal cancer is significantly elevated, which is 10-fold greater than that in the general western population.^2,3^ The increasing risk of developing cancer is strongly related to the duration, extent, and severity of inflammatory disease.^4^ A typical model mimicking this process is colitis-associated colorectal cancer (CAC), which is induced by combined administration of azoxymethane (AOM) and dextran sulfate sodium (DSS). Although the molecular mechanisms underlying transition from colitis to colorectal cancer remain elusive, a set of proinflammatory factors secreted by immune and non-immune cells has been shown to contribute to tumor growth in chronic intestinal inflammation.^5^

Granulocyte macrophage colony-stimulating factor (GM-CSF, also named CSF2) is one of hematopoietic cytokine family and its levels significantly elevated in the lesions of patients and rodents with colitis.^6,7^ In accordance with this, GM-CSF-deficient mice were more susceptible to DSS-induced colitis, as shown by more weight loss and severe diarrhea and by upregulation of proinflammatory mediator production.^8^ The role of GM-CSF in CAC development, however, is largely unknown. Recently, Wang et al.^9^ have reported that blockade of GM-CSF bioactivity using neutralizing specific monoclonal antibodies dramatically repressed CAC formation, through stimulating vascular endothelial growth factor (VEGF) release of colonic epithelial cells (CECs). Given that GM-CSF has a great impact on host intestinal immunity under physiological and pathological conditions,^10^ the regulation of immune microenvironment (e.g. MDSCs) in CAC settings by GM-CSF is deserved to be further addressed.

MDSCs represent a heterogeneous population of myeloid cells, which comprise immature macrophages, granulocytes, dendritic cells (DCs), and other myeloid cells at earlier stages of differentiation that can be identified in mice by expression of CD11b and Gr-1.^11^ Tumor-derived factors mobilize MDSCs from bone marrow into peripheral blood, and then MDSCs suppress anti-tumor immunity and thereby favor tumor growth.^12–14^ The inhibitory properties of MDSCs have been proposed to be attributed to the expression of inducible nitric oxide synthase (iNOS) and arginase-1 (Arg-1), which are both involved in the metabolism of l-arginine.^15^

Recently, massive accumulation of MDSCs in the lesions of inflammatory bowel disease (IBD) and CAC has been observed.^16,17^ These cells suppress cytotoxic T-cell responses and promote CAC development, whereas the regulation of MDSC generation and accumulation in this setting is still elusive. We hypothesized that GM-CSF plays an important role in MDSC accumulation in CAC, considering the function of this cytokine on MDSC generation and recruitment reported in several tumor models.^18^ Herein, we showed that MDSC accumulation was profoundly impaired in CSF2−/− mice subjected to CAC induction. Supplement of MDSCs from CAC-bearing wild-type (WT) mice into CSF2−/− littermates resulted in recurrence of CAC. Furthermore, the supernatants of tumor explant culture or GM-CSF alone enabled differentiation of hematopoietic progenitors into MDSCs. Blocking GM-CSF activity remarkably abrogated this effect.

## Materials and methods

### Mice

C57BL/6 WT and CSF2−/− mice were purchased originally from Jackson Laboratory (Bar Harbor, Maine). Animals were housed in specific pathogen-free conditions with an alternating light/dark cycle. All experiments were performed using 6- to 8-week-old male mice. Care, use and treatment of mice in this study were in strict agreement with international guidelines for the care and use of laboratory animals and approved by Animal Ethics Committee of Jilin University.

### Establishment of CAC model

**Figure 1.**
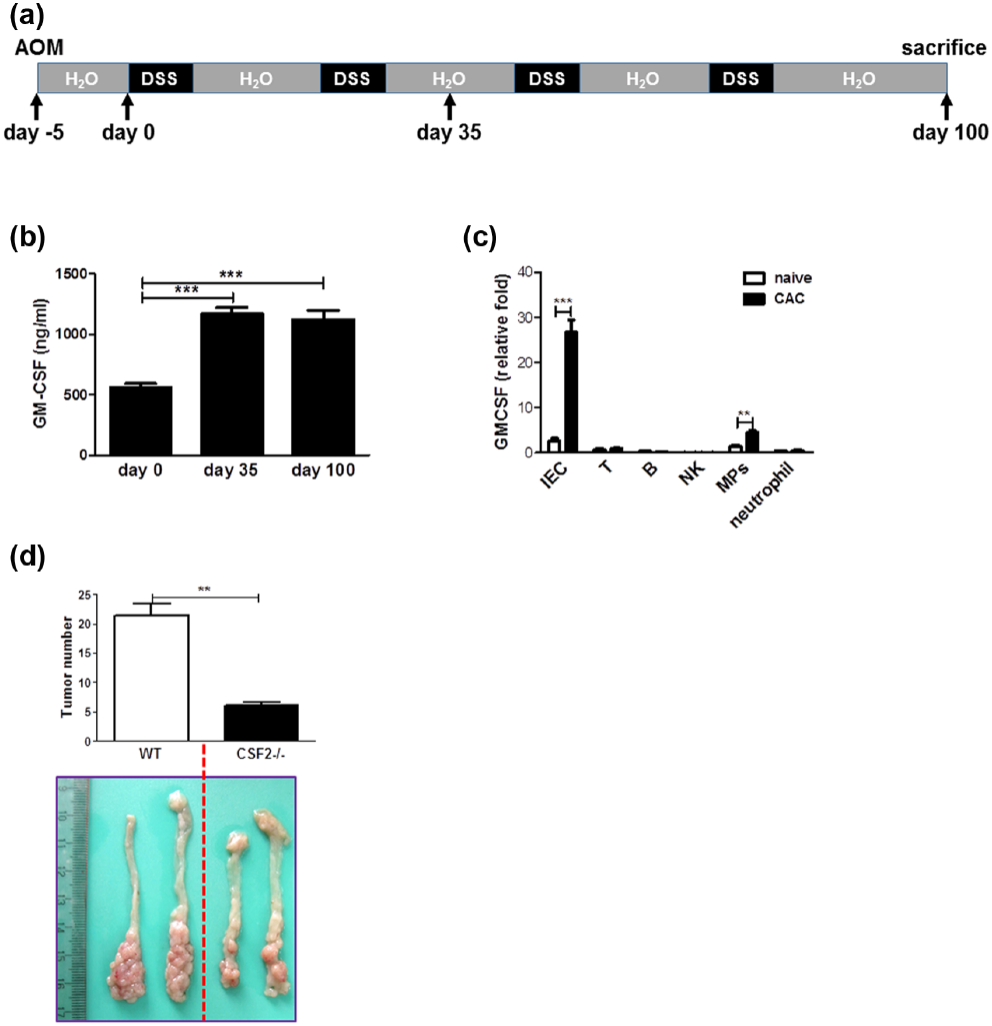
GM-CSF is required for CAC development. (a) Schematic overview of CAC regimen. (b) On days 35 and 100, colon explants were cultured ex vivo for 24 h. GM-CSF contents in the supernatants were examined by ELISA. (c) On day 100 after initiation of CAC induction, colonic epithelium and immune cell subsets residing in the LP of colon tissues were fractionated and GM-CSF expression was determined by quantitative RT-PCR. (d) CAC was induced in CSF2−/− mice and wild-type littermates as described in “Materials and methods.” Tumor number in the colon and rectum was counted. The data were pooled from three independent experiments. Each group consists of six to eight mice. **P < 0.01; ***P < 0.001.

CAC was induced according to classical protocols as described previously,^19^ with mild modification (Figure 1(a)). On day 1, mice were injected intraperitoneally with AOM (10 mg/kg; Sigma-Aldrich) and maintained on regular diet and water for 5 days. Mice then received water with 2% DSS (MW 36, 000-50, 000; MP Biochemicals) for 1 week. After this, mice were maintained on regular water for 2 weeks and subjected to three more DSS treatment cycles. On day 35 or 100, mice were sacrificed. Macroscopic tumors were counted. The clinical course of disease was followed daily by measurement of body weight and monitoring for signs of rectal bleeding or diarrhea.

### Immunohistochemistry

Colons were removed from mice and fixed in 10% neutral-buffered formalin solution and then embedded in paraffin, cut into tissue sections. Paraffin-embedded slides were deparaffinized and immersed in 80°C water bath in 10 mM sodium citrate buffer with 0.1% Tween 20 overnight for antigen unmasking. Slides were incubated with primary antibody against Gr-1 (BD Biosciences) and CD8α (eBiosciences) in phosphate-buffered saline (PBS) containing 1% bovine serum albumin (BSA) and 10% goat serum. Biotinylated secondary antibody (Dako) was added and incubated at room temperature for 1 h. Streptavidin–horseradish peroxidase (HRP) (BD Pharmingen) was added, and after 40 min the sections were stained with DAB (3,3′-diaminobenzidine) substrate and counterstained with hematoxylin.

### Isolation of CECs and lamina propria immune cell subsets

CECs were fractionated as described previously^20^ with the purity >80%, identified by CK-18 staining. For isolation of lamina propria (LP) immune cell subsets, the entire length of colon was opened longitudinally, washed with PBS, washed with PBS containing 100 µg/mL gentamycin, and cut into small pieces. The dissected mucosa was incubated with Ca^2+^, Mg^2+^-free Hank’s Balanced Salt Solution (HBSS) supplemented with 10% fetal bovine serum, 5 mM ethylenediaminetetraacetic acid (EDTA), 15 mM HEPES, 100 µg/mL gentamycin under shaking at 37°C for 20 min then repeated for four times. The supernatants containing CECs were depleted and the remaining was washed with HBSS, followed by digestion in RPMI 1640 medium containing 100 U/mL collagenase D (Roche Diagnostics) under shaking at 37°C for 1 h then repeated for two times. The total of digested supernatants was centrifuged at 800*g* for 10 min at 4°C and washed with PBS. The pellet was layered on a 40%/100% Percoll gradient (Pharmacia) and spun at 1800 r/min for 5 min to collect the mononuclear immune cell–enriched population at the 40%/100% Percoll interface.

### Flow cytometry

A single cell suspension prepared from colonic tissues as described above was fixed using PBS containing 1% paraformaldehyde and stained on ice for 25 min with antibodies in PBS containing 2% heat-inactivated fetal calf serum. The antibodies used were fluorescein isothiocyanate (FITC)-labeled anti-murine Gr-1 (RB6-8C5, eBioscience), and allophycocyanin-labeled anti-murine CD11b (M1/70, BD Pharmingen). Data collection and analysis were performed on a fluorescence-activated cell sorting (FACS) Calibur flow cytometer using CellQuest software (Becton Dickinson). For cell sorting, a single cell suspension from the colon was stained with antibodies and sorted on a FACS AriaII (Becton Dickinson). The antibodies used for sorting were those described above plus FITC-labeled anti-murine T-cell receptor (TCR)-β (H57-597, eBioscience), Ly-6G (1A8, eBioscience), CD19 (1D3. eBioscience) or CD3 (145-2C11, eBioscience), PE-labeled anti-murine CD49b (DX5, Biolegend), CD11c (N418, BD Pharmingen), or F4/80 (BM8, Biolegend).

### Mouse whole colon cultures

Colon tissue (200–300 mg) was washed in cold PBS containing penicillin and streptomycin and cut into small pieces (0.5 cm × 0.5 cm), which were cultured (three pieces per mouse) in 24-well flat bottom culture plates in serum-free RPMI 1640 medium (Gibco) at 37°C for 24 h. The culture supernatants were then centrifuged at 9000*g* at 4°C for 5 min and stored at −80°C until use.

### Suppression of polyclonal T-cell proliferation

FACS-sorted MDSCs were added in various amounts to 5 × 10^5^ splenocytes isolated from naïve C57BL/6 mice, in round-bottom 96-well plates. These cocultures were promptly stimulated with 5 µg/mL Con A for 72 h. Cultures were pulsed with 1 µCi/well [^3^H] thymidine for the last 16 h, and then the cells were harvested and counted by standard liquid scintillation. Inhibitors of Arg-1 and/or iNOS included *N*-hydroxy-l-arginine (NOHA; 100 µM), an inhibitor of iNOS and Arg-1 (Calbiochem), the specific Arg-1 inhibitor *N*-hydroxy-nor-l-arginine (nor-NOHA, 50 µM), and the NOS inhibitor l-*N*-iminoethyl-lysine (L-NIL; 5 µg/mL) were added from the beginning of the culture.

### Adoptive transfer of MDSCs

A total of 3 × 10^6^ FACS-sorted MDSCs or non-MDSCs (Gr-1^−^CD11b^+^) were intravenously injected into one CSF2-deficient mouse twice a week from the beginning of DSS treatment to the end of the experiments.

### Induction of MDSCs in vitro

Induction of Gr-1^+^CD11b^+^ cells from bone marrow progenitors was done as described previously,^21,22^ with mild modifications. In brief, bone marrow cells were obtained from the femurs and tibias. C-kit^+^lineage^−^ progenitors were sorted by FACS and cultured in 24-well plates in medium conditioned by the supernatants from colon explants or recombinant murine GM-CSF (50 ng/mL; Peprotech), supplemented with 10% fetal calf serum (Gibco) and interleukin (IL)-4 (20 ng/mL; Peprotech). In some experiments, purified neutralizing anti-GM-CSF monoclonal antibody (1 µg/mL; Biolegend) was added into the culture. Cells were collected 7 days later, and the proportion of Gr-1^+^CD11b^+^ cells was analyzed by flow cytometry.

### Cytokine analysis

GM-CSF was examined in whole colon culture supernatants by ELISA kits obtained from R&D Systems, according to the manufacturer’s instructions.

### l-Arginine detection

High-performance liquid chromatography (HPLC)-electrochemical detector was performed using an ESA CoulArray Model 540, with an 80 × 3.2 column with 120A pore size as described previously.^23^ The supernatants were deproteinized and derivatized with 0.2 M ophthalaldehyde/β-mercaptoethanol. A total of 50 µL was injected per sample. Standards of l-arginine in methanol were run with each experiment.

### MDSC depletion

To deplete MDSCs, mice received intraperitoneal (i.p.) injections of 250 µg of purified monoclonal anti-Gr-1 antibody (RB6-8C5, Biolegend) or a control isotype on days −3 and −2 before initiation of DSS drinking, then twice a week (days 2 and 5 each week) throughout the entire experimental period to ensure depletion. Depletion efficiency was confirmed at the end of experiments using flow cytometry (>80% MDSCs were depleted).

### Quantitative reverse transcriptase polymerase chain reaction

RNA extracted from colon epithelial cells or cell lines was reverse-transcribed into complementary DNA (cDNA) using the SuperScriptIII First Strand cDNA synthesis system (Invitrogen). cDNA was synthesized from 0.5 µg RNA using random hexamer primers and SuperScriptIII (Invitrogen). Real-time reverse-transcriptase-polymerase chain reaction (RT-PCR) was performed on a Bio-Rad iCycler to quantify messenger RNA (mRNA) levels. The primers are as follows: GM-CSF—sense: 5′-TCT CAG CAC CCA CCC GCT CA-3′, anti-sense: 5′-GCC CCG TAG ACC CTG CTC GAA-3′; Arg-1—sense: 5′-CTC CAA GCC AAA TAC AAG A-3′, anti-sense: 5′-AGG AGC TGT CAT TAG GGA CAT C-3′; iNOS—sense: 5′-GTT CTC AGC CCA ACA ATA CAA GA-3′, anti-sense: 5′-GTG GAC GGG TCG ATG TCA C-3′; and glyceraldehyde 3-phosphate dehydrogenase (GAPDH)—sense: 5′-TCT TGG GCT ACA CTG AGG AC-3′, anti-sense: 5′-CAT ACC AGG AAA TGA GCT TGA-3′. All reactions were performed in triplicate. The data were analyzed using Q-Gene software and expressed as fold change mean normalized expression (MNE) from control value. MNE is directly proportional to the amount of RNA of the target gene relative to the amount of RNA of the reference gene, GAPDH.

### Statistical analysis

Data are presented as mean ± standard deviation (SD). Student’s *t*-test (two-tailed) was used to determine significance, with *P* < 0.05 considered significant. Statistics were performed using SPSS 10.0 for Macintosh, and graphs were made on Deltagraph (SPSS).

## Results

### GM-CSF is indispensable for CAC development

First, we performed the ex vivo culture of colon explants of mice suffering from CAC and detected GM-CSF levels in the supernatants. The results showed a significant increase in GM-CSF contents in the colonic tissues (Figure 1(b)). This increase was observed as early as on day 35 after initiation of CAC induction (Figure 1(b)), indicating that GM-CSF may be a key factor linking inflammation and cancer. Furthermore, in accordance with a previous study,^9^ GM-CSF was predominantly secreted by CECs as well as mononuclear phagocytes (MPs) residing in the LP of the colon, although the abundance of this cytokine in the latter population was much lower than that in CECs (Figure 1(c)). In contrast, other immune cell subsets including T, B, natural killer (NK), and neutrophils expressed a bit of GM-CSF and its expression did not change following CAC induction (Figure 1(c)).

To precisely define the role of GM-CSF in CAC development, CAC model was established in CSF2−/− mice and WT littermates. We found that ablation of GM-CSF dramatically inhibited CAC formation (Figure 1(d)), suggesting clearly a CAC-promoting role of this cytokine.

### MDSC massively accumulates in lesions and supports CAC development

**Figure 2.**
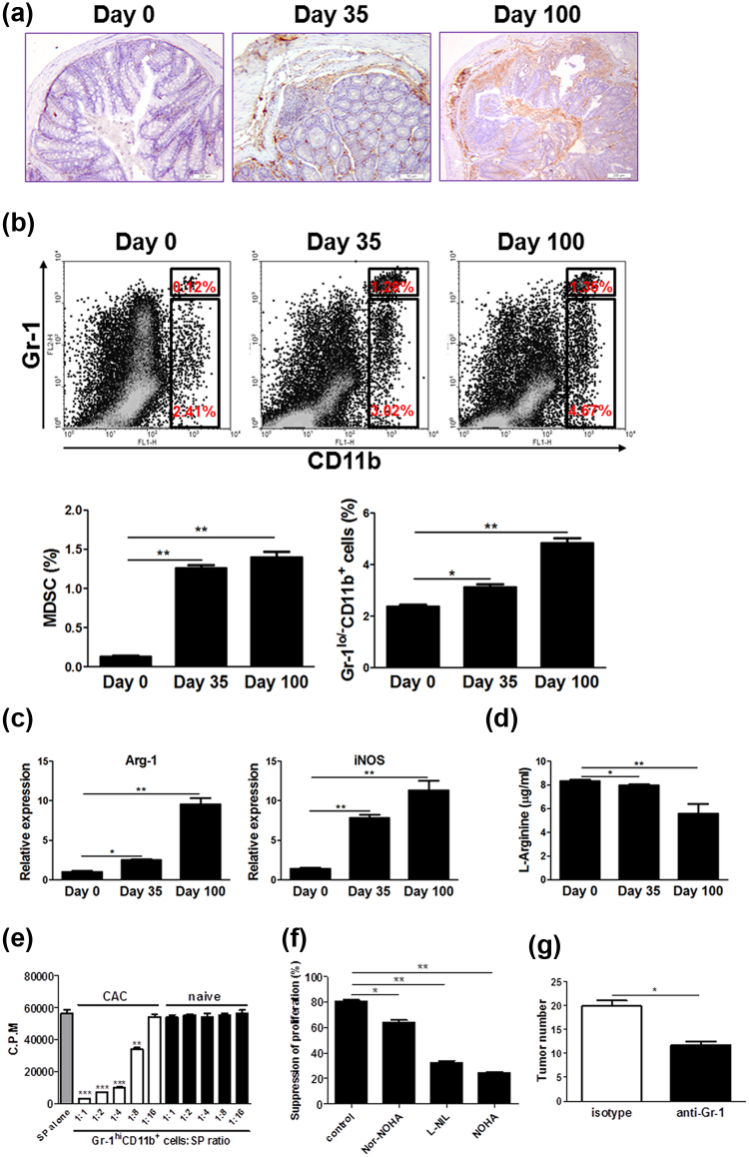
MDSCs accumulate increasingly in CAC lesions and have a critical role in CAC development. On days 0, 35, and 100 after initiation of CAC induction, the following experiments were performed. (a) Colon tissues were dissected and fixed with formalin, then processed conventionally. MDSCs were detected by staining with anti-Gr-1 antibody (brown). The representative images from three independent experiments were shown. Scale bar: 200 µm. (b) Immune cells in the LP of the colon were isolated and the percentages of MPs (Gr-1^lo/−^CD11b^+^) and MDSCs (Gr-1^hi^CD11b^+^) were examined by flow cytometry. The representative plots from three independent experiments were shown. (c) LP immune cells were isolated as described in “Materials and methods.” Gr-1^+^CD11b^+^ cells in LP were purified by FACS sorting. Arginase 1 (Arg-1) and inducible nitric oxide synthase (iNOS) expression in Gr-1^+^CD11b^+^ cells was determined by quantitative RT-PCR. (d) The pieces of colon tissues were dissected and cultured in serum-free medium ex vivo for 24 h. The supernatants were collected and l-arginine contents were examined by HPLC analysis. (e) Gr-1^+^CD11b^+^ cells were isolated from the colonic LP of CAC-bearing mice and naïve littermates and cocultured with splenocytes (SP) from naïve C57BL/6 mice at titrated ratios indicated, with stimulation of Con A (5 µg/mL) for 72 h. The proliferation was evaluated by [^3^H]-thymidine incorporation. The data were pooled from three independent experiments. (f) The coculture of Gr-1^+^CD11b^+^ cells was isolated from the colonic LP of CAC-bearing mice and splenocytes at 1:4 ratios were preincubated with Nor-NOHA (50 µM), L-NIL (5 µg/mL) and NOHA (100 µM), respectively, and stimulated with Con A (5 µg/mL) for 72 h. The proliferation was evaluated by ^3^[H]-thymidine incorporation. (g) Mice subjected to CAC induction were injected intraperitoneally with anti-Gr-1 antibody or isotypes according to the regimen as described in “Materials and methods.” Tumor number in the colon and rectum was counted. The data were pooled from three independent experiments. Each group consists of six to eight mice. *P < 0.05; **P < 0.01; ***P < 0.001 versus controls.

It is well known that GM-CSF enables MDSC generation and accumulation in several tumor models, so we assumed that MDSCs may be intermediates linking GM-CSF and CAC. To address this issue, first we detected MDSC abundance in lesions of the colon of mice subjected to CAC. Massive accumulation of MDSCs was observed in the inflamed tissues following CAC, which was confirmed by immunohistochemistry (Figure 2(a)) and flow cytometry (Figure 2(b)), respectively. Intriguingly, the frequencies of MPs (Gr-1^lo/^-CD11b^+^) residing in LP also increased significantly following CAC induction (Figure 2(b)), which coincided with the elevation of GM-CSF levels in lesions. To characterize the feature of MDSCs, these cells were sorted from lesions on days 0, 35, and 100 after initiation of CAC induction, respectively, and Arg-1 as well as iNOS expression was examined. The results showed compared to those on day 0, MDSCs on days 35 and 100 contained high amounts of Arg-1 and iNOS (Figure 2(c)). Consistently, l-arginine contents in the colonic tissues dramatically decreased at the same timepoints (Figure 2(d)). These data suggest massive accumulation of MDSC during the progression from colitis to cancer.

Furthermore, we evaluated the immune-suppressive capacity of MDSCs. We found that these cells isolated from the lesions of CAC-bearing mice pronouncedly suppressed the proliferation of splenocytes with stimulation of Con A (Figure 2(e)). This effect was obvious even at the ratio of 1:8. In sharp contrast, Gr-1^+^CD11b^+^ cells isolated from naïve C57BL/6 mice had no effect on splenocyte expansion. To define the role of Arg-1 and iNOS in the suppressive function of MDSCs, appropriate inhibitors were added into the coculture. The results showed that both Arg-1 and iNOS were required for the suppressive effects of MDSCs on lymphocyte expansion (Figure 2(f)). Moreover, the suppression appeared more significant when iNOS inhibitor was added (Figure 2(f)).

To directly define MDSC role in CAC development in vivo, this population was depleted by injection of neutralizing anti-Gr-1 antibodies into mice, and then CAC was established. The results showed that depletion of MDSCs remarkably repressed CAC formation (Figure 2(g)). These findings suggest that MDSC accumulation is a key event for CAC pathogenesis.

### MDSC accumulation accounts for the CAC-promoting function of GM-CSF

**Figure 3.**
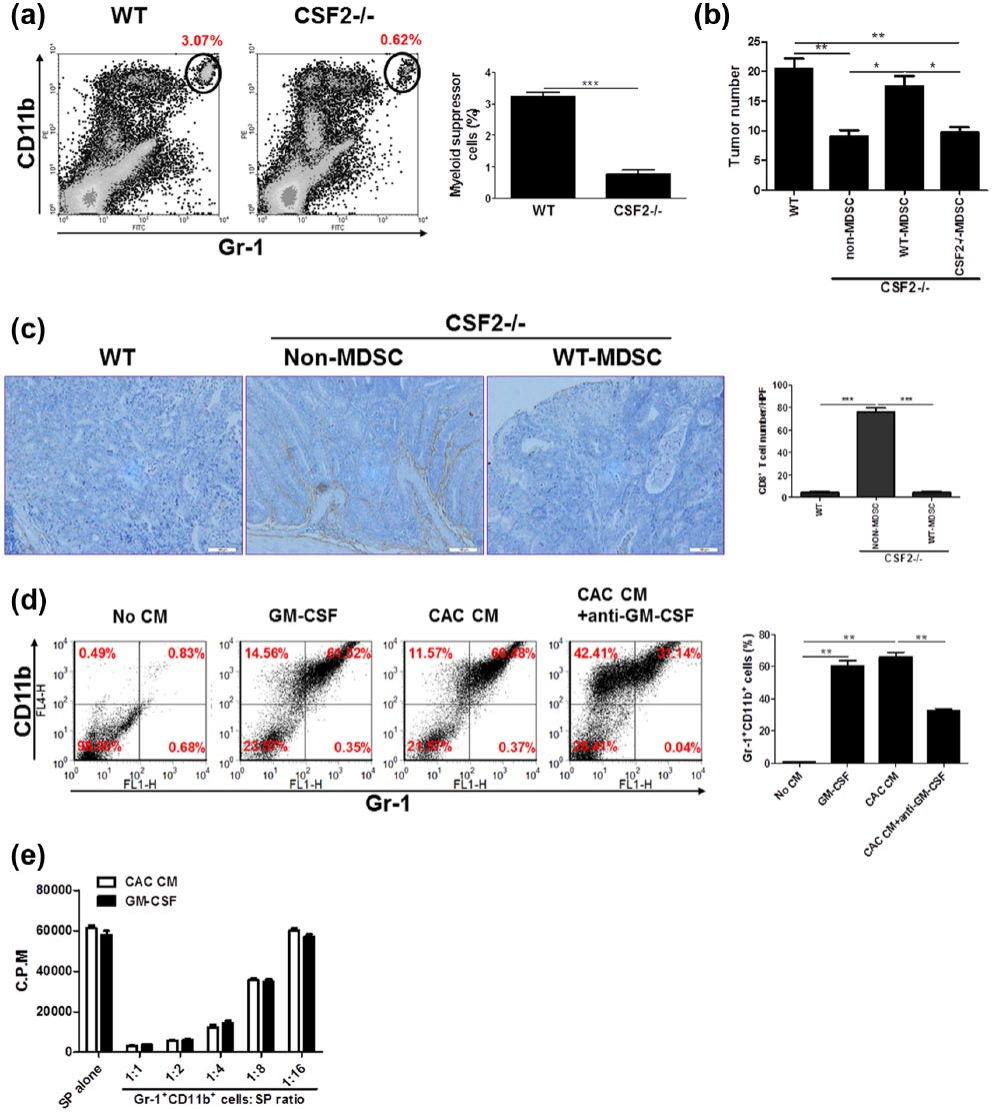
The CAC-promoting role of GM-CSF is via inducing/recruiting MDSCs. (a) CAC was induced in CSF2−/− mice and wild-type littermates and the percentages of Gr-1^+^CD11b^+^ cells in the LP of the colon were examined by flow cytometry. The representative plots from three independent experiments were shown. (b and c) Wild-type and CSF2−/− mice were transfused intravenously with MDSCs or non-MDSC (3 × 10^6^/mouse), respectively, followed by CAC induction. Tumor number in the colon and rectum was counted. (b) Colon tissues were dissected and processed conventionally. CTLs were detected by staining with anti-CD8α antibody (brown). The representative images from three independent experiments were shown. (c) Scale bar: 100 µm. (d) c-kit+lineage- hematopoietic progenitors isolated from bone marrow of C57BL/6 mice were treated with the supernatants of colon tissues of CAC-bearing mice (CAC CM) and recombinant murine GM-CSF (50 ng/mL) for 7 days. Neutralizing anti-GM-CSF monoclonal antibody (1 µg/mL) was added into the culture. The percentages of Gr-1^+^CD11b^+^ cells were examined by flow cytometry. The representative plots from three independent experiments were shown. (e) Gr-1^+^CD11b^+^ cells were pooled from CAC CM or GM-CSF-treated culture outputs and cocultured with splenocytes (SP) from naïve C57BL/6 mice at titrated ratios indicated, with stimulation of Con A (5 µg/mL) for 72 h. The proliferation was evaluated by [^3^H]-thymidine incorporation. The data were pooled from three independent experiments. Each group consists of six to eight mice. *P < 0.05; **P < 0.01; ***P < 0.001.

To uncover the causal relationship between MDSC accumulation and GM-CSF elevation, we detected MDSC number in lesions of CSF2−/− mice subjected to CAC. The results showed that the percentages of MDSC decreased significantly in AOM/DSS-treated CSF2−/− mice compared with WT counterparts (Figure 3(a)). Furthermore, we transfused MDSC from lesions of CAC-bearing WT mice into CSF2−/− mice. This supply indeed led to recurrence of CAC, strongly suggesting that GM-CSF promoted CAC development by regulating MDSCs (Figure 3(b)). Of note, transfusion of the equal quantity of non-MDSC (Gr-1^−^CD11b^+^) or MDSCs from lesions of AOM/DSS-induced CSF2−/− mice had no effect (Figure 3(b)).

Given that the tumor-promoting function of MDSCs is largely due to blocking cytotoxic CD8^+^ T-cell infiltration into lesions, we detected this lymphocyte population infiltration in WT and CSF2−/− mice subjected to AOM/DSS. As a result, the infiltration of CD8^+^ T cells into the lesions of WT mice was significantly suppressed (Figure 3(c)). Furthermore, transfusion of WT MDSCs, instead of non-MDSCs, into CSF2−/− mice led to a dramatic decrease of CD8^+^ T-cell frequency in the lesions (Figure 3(c)), which establish a causal relationship between MDSC function and CTL infiltration.

To further define a direct function of GM-CSF on MDSC generation, hematopoietic progenitors (c-kit^+^lineage^−^) were fractionated from bone marrow and treated with GM-CSF or the supernatants of colon explant culture in vitro (here named CAC CM). The results showed that GM-CSF alone sufficiently induced progenitors to differentiate into Gr-1^+^CD11b^+^ cells, which resembled CAC CM (Figure 3(d)). These cells phenocopied MDSCs, as shown by the suppressive properties to lymphocyte proliferation (Figure 3(e)). Importantly, blockade GM-CSF bioactivity in CAC CM drastically abrogated this effect (Figure 3(d)), indicating that GM-CSF represents a key mediator for MDSC accumulation in CAC.

## Discussion

It is well established that a variety of proinflammatory cytokines, which are produced by epithelial and stromal cells in tumor microenvironment, are involved in CAC pathogenesis by regulating neoplastic cell proliferation and/or forming tumor-promoting niches.^24^ As one important member of proinflammatory cytokine family, the role of GM-CSF in CAC has recently been uncovered that it promoted CAC development by eliciting epithelial VEGF release.^9^ However, the axis of GM-CSF-VEGF could not fully explain the tumor-promoting effect of GM-CSF. There remain other mechanisms to be addressed. We here provide evidence that MDSC accumulation is involved in the protumor effect of GM-CSF. That is, GM-CSF regulates MDSC generation and recruitment into colonic lesions and thereby facilitates CAC development. Therefore, it appears conceivable that GM-CSF favors inflammatory colon tumorigenesis in immune-dependent and independent manners.

Although GM-CSF clearly regulates hematopoiesis as a growth factor, its role as immunomodulatory cytokine has become increasingly appreciated. Depending on the setting, these effects can either promote or suppress cellular immune responses. In studies of implantable tumor models, GM-CSF has been linked to the generation of Gr-1^+^CD11b^+^ cells with immunosuppressive characteristics.^25–28^ For example, GM-CSF-knockin melanoma cells enable the systemic expansion of Gr-1^+^CD11b^+^ cells and inhibit memory CD8^+^ T cells. Knockdown of GM-CSF in tumor cell lines has been shown to alter the subsets distribution of Gr-1^+^CD11b^+^ myeloid cells when implanted in mice, mainly reducing the Gr-1^low^ and Gr-1^int^ populations which are the most suppressive in these models.^26^ Here, we show that GM-CSF is produced in vivo in CAC that is characterized by the prominent infiltration of suppressive Gr-1^+^CD11b^+^ cells.

Of note, a set of cytokines and growth factors other than GM-CSF has been implicated in the pathophysiology of MDSCs in several mouse models including IL-1β, IL-6, and VEGF.^29^ It is interesting that the production of these cytokines significantly increased in CAC niches.^30–32^ Although the relationships between these cytokines and MDSC accumulation in CAC remain to be addressed, we propose that GM-CSF plays a dominant role in MDSC accumulation in this model because GM-CSF deficiency renders 80% reduction of MDSCs in the lesions. In terms of other members of colony-stimulating factors (i.e. G-CSF and M-CSF), their levels have not been detected in this study, but previous studies have shown that the increase in their production in CAC lesions was observed at the late stage instead of the early stage of CAC.^9^ It is thus reasonable that G-CSF and M-CSF may be dispensable for the early-stage accumulation of MDSCs in CAC lesions and possibly be involved in the late-stage accumulation of these cells. Recently, another proinflammatory mediator prostaglandin E2 (PGE2) has been demonstrated to be linked to MDSC accumulation in CAC model.^17^ Therefore, we can make a conclusion that GM-CSF, with synergistic action of other factors (e.g. PGE2), recruits MDSCs into lesions and eventually promotes CAC growth.

Furthermore, we have shown that compared with WT MDSCs, the equal number of MDSCs from CSF2−/− mice cannot recover CAC in CSF2−/− recipients subjected to AOM/DSS. This indicates that the suppressive capacity of MDSC was seriously blunted in the absence of GM-CSF. In support of this viewpoint, GM-CSF alone is sufficient to induce the generation of immunosuppressive MDSCs in vitro, which is consistent with previous observations in a spontaneous model of pancreatic ductal adenocarcinoma.^33^ It should be noted that CSF2−/− mice have relatively normal hematopoiesis in bone marrow.^34,35^ So, the decrease in MDSC number in lesions of CSF2−/− mice appears to be due to the impairment of differentiation of progenitors into MDSCs and/or the inhibition of MDSC recruitment into lesions.

In conclusion, our study confirms the pathogenic role of GM-CSF in CAC development. GM-CSF favors tumor-permissive microenvironment by inducing MDSC generation and recruiting them into colonic tissues. MDSCs thereafter actively suppress anti-tumor immune responses and support CAC growth. Considering a direct function of MDSCs on cancer cells^36^ or their effect on angiogenesis,^37^ further investigations in the details of MDSC function on CAC in the future are warranted. Overall, our findings shed new insights on the understanding of mechanisms underlying CAC pathogenesis. Targeting GM-CSF or blocking MDSC generation and/or recruitment using appropriate inhibitors may represent new regimens for the treatment of CAC in clinic.
